# Guanidine–Curcumin Complex-Loaded Amine-Functionalised Hollow Mesoporous Silica Nanoparticles for Breast Cancer Therapy

**DOI:** 10.3390/cancers14143490

**Published:** 2022-07-18

**Authors:** Thimma Mohan Viswanathan, Kaniraja Chitradevi, Azar Zochedh, Ramakrishnan Vijayabhaskar, Sureba Sukumaran, Selvaraj Kunjiappan, Nachimuthu Senthil Kumar, Krishnan Sundar, Ewa Babkiewicz, Piotr Maszczyk, Thandavarayan Kathiresan

**Affiliations:** 1 Department of Biotechnology, Kalasalingam Academy of Research and Education, Krishnankoil 626126, India; viswabiotech9@gmail.com (T.M.V.); chitradevik11@gmail.com (K.C.); azar2411@gmail.com (A.Z.); surebasukumaran95@gmail.com (S.S.); selvaraj.k@klu.ac.in (S.K.); sundarkr@klu.ac.in (K.S.); 2 Department of Surgical Oncology, Meenakshi Mission Hospital and Research Centre, Madurai 625107, India; drvijayabhaskar@gmail.com; 3 Department of Biotechnology, Mizoram University, Aizawl 796004, India; nskmzu@gmail.com; 4 Department of Hydrobiology, Faculty of Biology, University of Warsaw, 02-089 Warsaw, Poland; ewa.babkiewicz@wp.pl

**Keywords:** anticancer, caspases, drug-loading capacity, drug release kinetics, nanomedicine

## Abstract

**Simple Summary:**

The combination of guanidine carbonate and curcumin-loaded hollow mesoporous silica nanoparticles (GuC-HMSNAP) can be used as a therapeutic and to induce MCF-7 cell death. Due to its biological safety and high drug loading capacity, HMSNAP is becoming an increasingly important nanocarrier for cancer research. The features of drug-loaded nanocarriers are significantly altered particle size, pore size, surface area, and pore volume, confirming that a significant amount of drugs can be loaded in the nanocarriers while simultaneously allowing the maximum amount of drugs to be released from the nanocarriers. Here, guanidine-mediated apoptosis is analysed through western blotting; the results suggest that drug complexes result in downregulation of phosphorylation in Ser471 of Akt, Ser259 of c-Raf, and Ser241 of PDK1, upregulation of phosphorylation in GSK-3β Ser9, cleaved caspases, and cleaved PARP, which then partially induces intrinsic cell death in MCF-7. As a whole, our results demonstrate that GuC-HMNSAP is an efficient nanocarrier for effectively inducing cancer cell death.

**Abstract:**

The current study focuses on developing a tumour-targeted functionalised nanocarrier that wraps hollow mesoporous silica nanoparticles. The guanidine carbonate and curcumin are immobilised on the surface of 3-aminopropyl-triethoxy silane (APTES)-decorated hollow mesoporous silica nanoparticles (HMSNP), as confirmed through XPS and NMR analysis. XPS analysis demonstrates that the shape of the hysteresis loops is modified and that pore volume and pore diameter are consequently decreased compared to control. Guanidine (85%) and guanidine–curcumin complex (90%) were successfully encapsulated in HMSNAP and showed a 90% effective and sustained release at pH 7.4 for up to 72 h. Acridine orange/ethidium bromide dual staining determined that GuC-HMNSAP induced more late apoptosis and necrosis at 48 and 72 h compared with Gu-HMNSAP-treated cells. Molecular investigation of guanidine-mediated apoptosis was analysed using western blotting. It was found that cleaved caspases, c-PARP, and GSK-3β (Ser9) had increased activity in MCF-7 cells. GuC-HMSNAP increased the activity of phosphorylation of oncogenic proteins such as Akt (Ser473), c-Raf (Ser249), PDK1 (Ser241), PTEN (Ser380), and GSK-3β (Ser9), thus inducing cell death in MCF-7 cells. Altogether, our findings confirm that GuC-HMNSAP induces cell death by precisely associating with tumour-suppressing proteins, which may lead to new therapeutic approaches for breast cancer therapy.

## 1. Introduction

Breast cancer is the most common cause of cancer incidence and death in women worldwide [1]. On the whole, current medications are unable to conquer breast cancer and reduce mortality. While chemotherapy is a widely accessible therapeutic modality, it generally does not target specific sites [2,3,4]. When a single chemotherapeutic agent is used, it frequently results in an unfavorable drug reaction and drug resistance [5,6,7]. To overcome these problems, more than one chemotherapeutic agent can be synergistically loaded into drug carriers for effective drug delivery systems (DDS) in the cancer microenvironment.

The production of massive drug carriers is one of the major targets of nanotechnology. Because of their large surface area, uniform pore size, and excellent drug loading capability, hollow mesoporous silica nanoparticles are particularly ideal carriers [7,8,9]. Amine functionalisation and polymer-coated HMSNPs improve nanocarrier drug loading, stability, and drug release capacity. Examples of amine molecules include APTES, while potential polymers include polyethyleneimine (PEI), polyethylene glycol (PEG), polycaprolactone (PCL), dextran, or chitosan [10,11]. Generally, HMSNPs are flexible nanocarriers for smart drug delivery systems [12]. The defined mesoporous structure is able to control and contribute to the sustained release of therapeutic molecules, in turn resulting in the release of a lower dosage and thereby reducing the side effects associated with overdosing. In addition, they effectively increase the cellular drug concentration and enhance the mode of drug action. These nanoparticles are involved in the management of effective cancer therapies through targeted drug delivery [13], continuous drug release [14], lesser side effects [15], and the ability to overcome multidrug resistance (MDR) [14,16]. Thus, the Food and Drug Administration (FDA) has given the green light for silica nanoparticles to be used in biomedical applications.

Cancer development is a multifaceted pathway, requiring the adoption of well-practiced cancer therapeutic strategies. As a consequence, a combination of therapies are required to synergistically counter cancer cells while causing no harm to normal cells [17]. Curcumin and its supplements have anticancer properties, as they prevent multiplication of malignant cell lines [18,19]. In particular, curcumin enters the mitochondria and endoplasmic reticulum through silica nanoparticles and induces cell death through activation of cleaved caspase 9, cleaved caspase 3, and cleaved PARP [20]. Guanidine functional groups are found in a large number of natural products and active pharmaceutical ingredients [21,22,23]. In order to recognise different receptors, the six-membered borazine ring of guanidine cations engages in a range of non-covalent interactions, including hydrogen bonds, electrostatic binding, and π-stacking associations [24]. The application of guanidine plays a major role in antimalarial [25], antihistamine, anti-inflammatory, and anticancer drugs [26,27], along with other fields of medicinal chemistry [28]. Guanidine is a molecular transporter which binds to inositol dimers of the plasma membrane that enhances the cell-penetrating capacity and then delivers a variety of compounds, including small molecules, peptides, proteins, and imaging agents [29]. Guanidine effectively interacts with adenine and thiamin phosphate residues in the DNA helix at the minor groove, thus damaging the DNA and inducing cell death [30]. In addition, the guanidine compound helps in the delivery of drug complexes to cancer cells while increasing the hydrophilic moiety, which later induces cytotoxicity against cancer cells. Several chemotherapeutic agents have been combined with guanidine compounds, for instance, doxorubicin [31], platinum complex [32], and dacarbazine [33], and are able to synergistically restrain different types of cancers. The goal of this guanidine complex is to test broad ranges of inhibition in neoplastic movement and growth in cancers such as cervical, colon, ovarian, and neuroendocrine tumours.

The use of guanidine carbonate, curcumin, and their combination loaded on HMSNP for decoration with APTES has yet to be attempted, as has their effective therapeutic delivery. In this study, we evaluate their interaction as well as their pH response, drug loading and release kinetics, and guanidine–curcumin complex-induced MCF-7 cell death. The present study focused on guanidine carbonate (Gu-HMSNAP) both singly and in combination with curcumin (GuC-HMSNAP)-loaded HMSNP coated with APTES, which were used to deliver drugs against a breast cancer cell line.

The following main questions are addressed in this research: (1) whether HMSNAP is less hazardous, what its effective drug loading capacity is, and its degree of pH-dependent sustained drug release in MCF-7 cells; (2) whether low concentrations of drug-loaded HMSNAP can cause cell death in MCF-7 cells when compared with free guanidine; and (3) whether guanidine–curcumin complex-loaded HMSNAP induces apoptosis and phosphorylation of oncogenic proteins in MCF-7 cells. Overall, our research shows that a combination of guanidine and curcumin (GuC-HMSNAP) has improved therapeutic potential, suggesting that it could be a superior alternative to current breast cancer treatments.

## 2. Experimental Procedure

### 2.1. Chemicals and Reagents

Tetraethyl orthosilicate (TEOS; ≥99.90%), 3-aminopropyl-triethoxy silane (APTES; ≥99.00%), cetyltrimethyl ammonium bromide (CTAB; ≥99.00%), dimethyl sulfoxide (DMSO; ≥99.70%), guanidine carbonate (≥99.00%), curcumin (≥99.50%), acridine orange (≥90.00%), and ethidium bromide (≥95.00%) were procured from Sigma-Aldrich, Bengaluru, India. Invitrogen, Carlsbad, California, USA and Gibco BRL, Waltham, MA, USA kindly provided Dulbecco’s Modified Eagle Medium (DMEM; ≥99.90%), fetal bovine serum (FBS; ≥99.90%), penicillin-streptomycin, and 0.25% trypsin EDTA and tetrazolium salt (3-(4, 5-dimethylthiazol-2yl)-2,5-diphenyltetrazolium bromide) (≥98.00%). All the experimental reagents and chemicals were prepared using 18 MΩ milli-Q water (Millipore system, Burlington, MA, USA). The other remaining reagents and chemicals were of cell culture grade and did not require any further purification.

### 2.2. Synthesis of HMSNAP

The synthesis of hollow mesoporous silica nanoparticles (HMSNP) was attained using the sol-gel emulsion method with slight modifications [34]. The catalysed ammonia was hydrolysed and condensed TEOS in an aqueous basic–ethanol medium using CTAB as a surfactant. Typically, CTAB (80 mg), TEOS (500 µL), and a 25.00% ammonia solution (500 µL) were dissolved in 40.50 mL of an ethanol–water mixture and stirred for 700 g at 30 °C to provide a white suspension. After a 3 h reaction, the resulting products were collected by centrifugation and washed with ethanol twice to remove residual organics and ammonia, then dried. The dried HMSNP was obtained through calcination at 800 °C for 6 h. For functionalisation of amines with nanoparticles, HMSNP (50 mg) and APTES (50 µL) were added to ethanol (50 mL) and incubated in an incubator shaker for 24 h. After incubation, the nanostructure was filtered and cleaned three times with ethanol and one time with milli-Q water and dried to form APTES-decorated HMSNAP.

### 2.3. Drugs Loading and Release

For drug loading and release, the above-incorporated HMSNAP (15 mg) was dissolved separately with an increased gradual concentration of guanidine (30 to 50 mM); a combination of guanidine (30 to 50 mM) and curcumin (30 mM) was dissolved in 10 mL of ethanol and later incubated for 24 h in a shaker. The free guanidine and curcumin were washed with ethanol and then dried. These nanostructures, which attained increased gradual absorption of guanidine- and curcumin-loaded HMSNAP, were used to identify the IC_50_ values of these drugs with MCF-7 cells. Fifteen mg of synthesised HMSN and HMSNAP were dissolved separately in a beaker containing 36 mM guanidine carbonate alone and a combination of 36 mM guanidine carbonate and 30 mM curcumin in 10 mL of ethanol for drug loading and release assays, then incubated for 48 h in an incubator shaker. The free guanidine carbonate and curcumin were predicted at regular intervals of every 6 h from 0 h to 48 h incubation, and its absorbance at 420 nm for guanidine release and 540 nm for guanidine–curcumin complex release using a fluorescent microplate reader (Biotek, Vermont, USA) was compared with the control. The amount of the guanidine carbonate and curcumin-loaded nanocarrier was determined using the formula (Drug loading = O.D value at 0 h − O.D value at different time intervals/O.D value at 0 h) × 100. 

Comprehensive drug release studies were conducted with 10 mL (2 mg mL^−1^) of 30 µM Gu-HMSNAP and a mixture of 20 µM guanidine and 30 µM curcumin-loaded GuC-HMSNAP dispersed in phosphate buffer saline (PBS) at three different pHs: 3, 6, and 7.4. At regular intervals of every 6 h and up to 66 h, around 200 μL of the sustained-release solution was transferred to a microplate reader at the wavelength of guanidine release at 420 nm and curcumin release at 540 nm. The measured solutions of this experiment were returned to the flask, and the volume of the sustained-release solution remained unchanged. 

### 2.4. Drug Release Kinetics

For drug release kinetic mechanisms of active drug release from dosage formulations, numerous kinetics models were established. Changes in drug release in both in vitro and in vivo behaviour may occur due to qualitative and quantitative changes in therapeutic product development, facilitating product development by minimising the need for bio-studies, which is always desirable. In order to establish the drug release kinetics and diffusion mechanism, the obtained in vitro guanidine-loaded HMSNAP and curcumin-loaded HMSNAP release data were used in five basic kinetic models (zero, first, Higuchi, Korsmeyer–Peppas, and Hixson–Crowell). The data for the release was calculated using the DD solver 1.0 program (Microsoft Excel plugin model on Windows platform); each drug release kinetic model illustrates a different drug release mechanism from the formed mesoporous silica nanoparticles. In particular, the diffusional exponent ‘n’ is a prime indicator of the drug release kinetic mechanism from the drug formulation in the Korsmeyer–Peppas model. When “*n* = 0.45”, the drug release is assessed by the Fickian diffusion; when “*n* = 0.89”, the drug release order represents the erosion mechanism/case II transport. When “0.45 *n* > 1.0”, the diffusion mechanism is non-Fickian. If it is determined that diffusion and drug release are substantial, no “*n*” values or kinetic data are calculated [35,36].

### 2.5. Characterisation of HMSNAP and Drug-Loaded HMSNAP

The prepared hollow mesoporous silica nanoparticles with loaded drugs before and after functionalisation were characterised by scanning electron microscopy (SEM), high resolution transmission electron microscopy (HRTEM), X-ray diffraction (XRD), Fourier transform infrared spectroscopy (FTIR), Dynamic Light Scattering (DLS), and Zeta Potential. The morphological changes and sizes of HMSNAP, Gu-HMSNAP, and GuC-HMSNAP were analysed using SEM (Evo18 Zeiss Munich, Germany). A specified quantity of prepared silica nanoparticles were individually dispersed with 1 mL of ethanol and sonicated for five minutes for Formvar-coated copper grid preparation. The micrographs were obtained using HRTEM (T12 tecnai, Hillsboro, OR, USA) at HT650 ES1000W t 120 kV. ImageJ software (National Institutes of Health, New York, USA) of HRTEM was used to measure the pore size of silica nanoparticles. The silica nanoparticles were dispersed in water, particle size was measured through dynamic light scattering (DLS), and the surface characterisation of zeta potential was analysed using an SZ-100 Nanoparticle Analyzer (Horiba, Kyoto, Japan). XRD patterns were obtained with a D8 Advance ECO XRD System (Bruker, Madison, WI, USA) equipped with a 3 kW X-ray tube with a copper target. Real-time multiple solid-state detector strips were used, and K alpha was maintained at 0.001°. FTIR (Nicolet 6700, Shimadzu, Nishinokyo, Japan) was performed by ensuring the loading of guanidine and guanidine-curcumin complex in HMSNAP, which were mixed with potassium bromide to form a pellet and scanned in the ranges of 4000–400 cm^−1^. The surface elemental analysis of the drug-loaded nanocarrier was analysed by X-ray photoelectron spectra using a VersaProbe III Scanning XPS Microprobe spectrometer (Physical electronics, Chanhassen, MN, USA) using an aluminium Kα X-ray source with an hv of 1486.6 eV. The nitrogen adsorption and desorption isotherms were analysed on a Quantachrome^®^ ASiQwin™ (Boynton Beach, FL, USA) automated surface area and pore-size analyser. The samples were degassed at 150 °C for 24 h under vacuum conditions. The specific surface areas from Brunauer–Emmett–Teller (SBET) and Barrett–Joyner–Halenda (BJH) analysis were used to determine the surface area, the pore-size distribution, and the pore volume. The guanidine and curcumin complex loaded with HMSNAP was assessed through ^1^H nuclear magnetic resonance (NMR). A Bruker AV 500 MHz spectrometer was used to measure the spectra using D_2_O, with tetramethylsilane representing the solvents and internal standard, respectively. Finally, TopSpin^®^ 4.0 software was used to examine the binding confirmation of drug molecules with nanoparticles. 

### 2.6. Cytotoxicity Assay

HEK 293 (Human embryonic kidney cell line) and MCF-7 (human breast adenocarcinoma cell lines) cells were sourced from the NCCS (National Centre for Cell Science), Pune, India, and maintained in DMEM containing 10% fetal bovine serum and 1% penicillin-streptomycin at 37 °C with 5% CO_2_ in a 95% humidified atmosphere. The in vitro cytotoxicity of HMSNAP and drug-loaded HMSNAP were studied against MCF-7 cells using MTT assay, as in the previously described method with minor changes [37]. Briefly, the HEK 293 and MCF-7 cells were grown in 96-well plates (1.5 × 10^4^ cells well^−1^) and incubated in a growth medium. HMSNAP, guanidine alone, Gu-HMSNAP, and GuC-HMSNAP were treated separately with HEK 293 cells at gradually increasing doses from 5 to 400 µg concentration for 24 h and MCF-7 cells at gradually increasing doses of 5 to 50 µM and incubated for 24, 48, and 72 h with a serum (0.5%) deprivation medium. After respective time intervals, the medium was replaced and washed with PBS, then 10 µL of MTT solution was added to each well and incubated for 4 h. The MTT was reacted with live cells of dehydrogenase reductase to produce indissoluble formazan crystal. The MTT containing media was removed, then 100 µL of DMSO was added into each well to form purple formazan crystal and each well was mixed properly. The absorbance was taken at 595 nm in a 96-well plate reader. The HEK 293 and MCF-7 cells were incubated without any treatment, as they were considered the control. The experiments were repeated three times, with results expressed as the percentage (%) of control cells. The percentage of cell proliferation and its inhibition were estimated with the following Equations (1) and (2):

Proliferation [%] = (A_sample_/A_control_) × 100 (1)

Inhibition [%] = 100% of proliferation(2)

The IC_50_ values of each drug and drug-loaded nanoparticles were calculated using Graph Pad Prism 5.

### 2.7. Evaluation of Apoptosis Using Acridine Orange/Ethidium Bromide (AO/EtBr) Staining

Evaluation of guanidine–curcumin complex-induced apoptosis was assessed using AO/EtBr dual staining, as described previously [38]. The Gu-HMSNAP-treated and GuC-HMSNAP-treated MCF-7 cells were maintained for 24, 48, and 72 h. The cells were then trypsinised, pelleted, and dissolved in 1 mL of PBS with 100 µL each of AO (50 µg mL^−1^) and EtBr (30 µg mL^−1^). Afterwards, the cells were viewed under an inverted fluorescence microscope using an FITC filter (Carl Zeiss, Oberkochen, Germany) to study the morphology of apoptotic and healthy cells. The percentages of live and dead cells were estimated with various randomly selected fields.

### 2.8. Western Blot Analysis

The MCF-7 cells treated with guanidine carbonate alone and with drug-loaded HMSNAP (Gu-HMSNAP and GuC-HMSNAP)-persuaded proteins were analysed through western blotting. The MCF-7 cells were cultivated in 50 mm petri plates and treated with an IC_50_ concentration of 35 µM free guanidine carbonate, 30 µM Gu-HMSNAP, and a combination of 25 µM guanidine carbonate and 30 µM curcumin-loaded GuC-HSMNAP for 24 h and 48 h incubation. After incubation, the MCF-7 cells were collected and lysed in a 50 mM Tris buffer containing protease inhibitor cocktails (Roche, Switzerland). The lysates were sonicated and then centrifuged at 12,000 rpm at 4 °C for 10 min. The proteins were measured with the Bradford method, and 50 µg of each sample was loaded in an SDS-PAGE run with 80 V for 2 h. The separated proteins were transferred into a nitrocellulose membrane through a semidry western blotting apparatus (Amersham Bioscience, Piscataway, NJ, USA). All of the antibodies were procured from Cell Signaling Technology (Danvers, MA, USA). Using 5% casein, the membrane was blocked and incubated with the respective primary antibodies at room temperature, followed by incubation of the respective HRP-linked secondary antibodies (Santa Cruz, CA, USA) for 1 h. A Luminglo solution (Thermo Scientific, Rockford, IL, USA) was used to identify the appearance of immunoreactive bands of the various proteins. A densitometric scanner (Bio-Rad, Hercules, CA, USA) was used to measure the phosphorylation and immunoreactive bands. Then, the blot was washed and reprobed with anti-β-actin as a loading control to ensure that an equivalent quantity of samples was loaded into each well. The presented data with similar results were representative of two independent experiments.

### 2.9. Statistical Analysis

All the experiments were performed in triplicate, and the experimental data are presented as standard error ± mean. A one-way analysis of variance (ANOVA) test was performed to assess the results between the control and all treated samples (*p* < 0.05).

## 3. Results and Discussion

### 3.1. Formation of Drug Complex, Synthesis and Characterisation of HMSNAP

**Figure 1:**
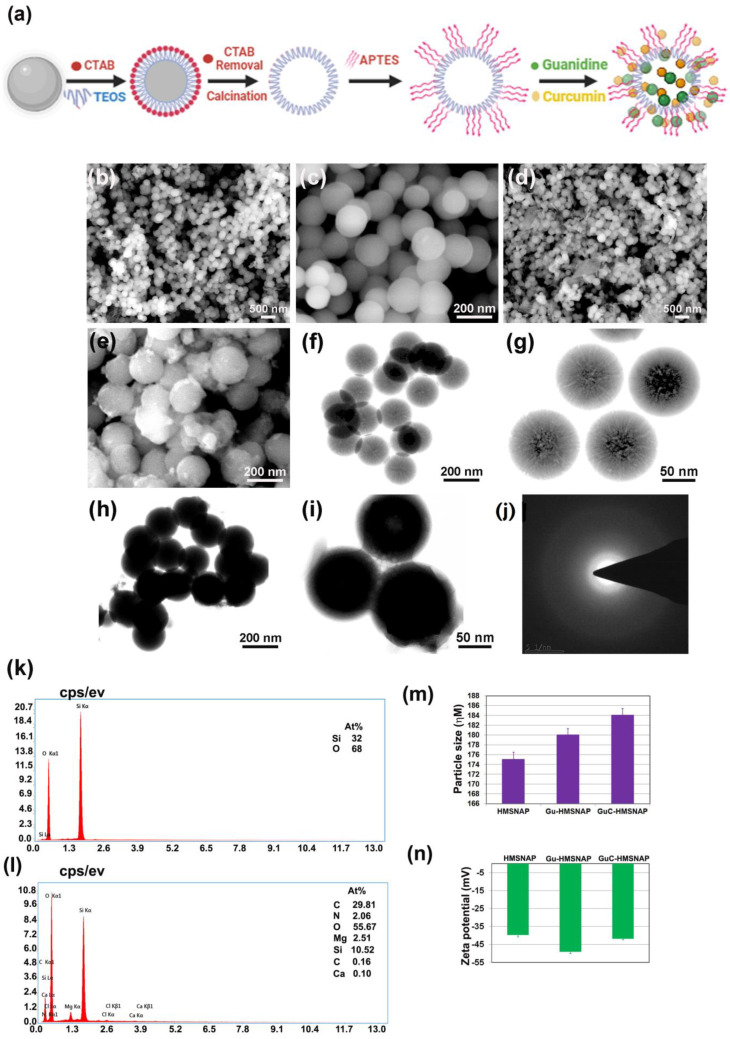
Schematic illustration of the synthesis of HMSNAP and encapsulation of guanidine alone and a combination of guanidine-curcumin complex as cargo with the nanocarrier (**a**). The surface morphology of HMSNAP (**b**,**c**) and guanidine-curcumin complex cargo with HMSNAP observed by SEM (**d**,**e**), TEM images of HMSNAP (**f**), and drug-loaded HMSNAP (**g**–**i**). SAED pattern of drug-loaded HMSNAP (**j**), EDX spectrum image of HMSNAP and drug-loaded HMSNAP (**k**,**l**), and DLS and Zeta potential graph of HMSNAP and drug-loaded HMSNAP (**m**,**n**).

Guanidine carbonate is a highly reactive organic compound, and is greatly shared with other molecules through hydrogen bonding and the Vander Waals force. Figure 1a illustrates how the guanidine and guanidine–curcumin complex binds with the nanocarrier HMSNAP through APTES decoration. The different concentrations of guanidine and the combination of guanidine–curcumin complex were loaded separately in synthesised HMSNAP. The surface morphology and internal composition of HMSNAP and drug-loaded HMSNAP were examined through SEM and TEM, as shown in Figure 1b–i. The surface of HMSNAP was smooth, whereas the surface of the drugs loaded with HMSNAP was rough because of the cargo on its carrier (see Figure 1b–d). The HMSNAP pores remain open and the predicted pore size was ~10 nm. However, the Gu-HMSNAP and GuC-HMSNAP pores were closed and densely concentrated because of drugs loaded in the nanocarrier (see Figure 1e–i). In addition, the electron diffraction (SAED) pattern of synthesised HMSNAPs revealed that mesoporous silica nanoparticles are amorphous in nature (Figure 1j). The EDAX study revealed that HMSNAP contained Si-32% and O-68%, while Gu-HMNSAP enclosed Si-22.13%, O-59.34%, N-3.1%, and GaC-HMSNAP together with Si-10.52%, O-55.67%, C-29.81%, Mg-2.51%, N-2.06%, and Ca-0.10% (Figure 1k,l). The elements of EDAX representing Si and O indicate silica and APTES, respectively. The N and C confirm that guanidine and curcumin are loaded on GuC-HMSNAP. 

### 3.2. Analysis of Nanocarrier Size, Surface Charge, and Functional Compounds 

In DLS analysis, the particle size of HMSNAP showed a 175 nm diameter; both Gu-HMSNAP and GuC-HMSNAP increased in size by 181 nm and 184 nm, respectively (Figure 1m). The increase in size of the nanocarrier could be attributed to the explicit volume of drugs loaded in both the hollow and mesopore of HMSNAP. Therefore, a higher quantity of guanidine–curcumin complex was loaded when compared with the nanocarrier loaded with guanidine alone. The zeta potential of the mesoporous silica nanocarrier is evaluated as shown in Figure 1n. The aminopropyl density of HMSNAP, Gu-HMSNAP, and GuC-HMSNAP increases significantly after APTES coating in HMSNP. The Si-O charges are highly exposed in silica nanoparticles due to the –NH2 function. Therefore, the HMSNAP, Gu-HMSNAP, and GuC-HMSNAP value of the zeta potential was negative. The surface charges of HMSNAP, Gu-HMSNAP, and GuC-HMSNAP were −39.6, −48.9, and −41.7 mV, respectively. The zeta potential of the guanidine and guanidine–curcumin complex-loaded nanocarriers gradually increased depending on the drugs loaded on the surface. In typical silica nanoparticles the zeta potential range is −28 to −61 mV [39], and the increase of zeta potential depends on the increased size of nanoparticles [40]. The zeta potential of the sol-gel emulsion of HMSNAP with −40 to −50 mV had good stability and prolonged drug release inside the cells [41].

### 3.3. XRD and FTIR Analysis of HMSNAP and Drug-Loaded HMSNAP

**Figure 2:**
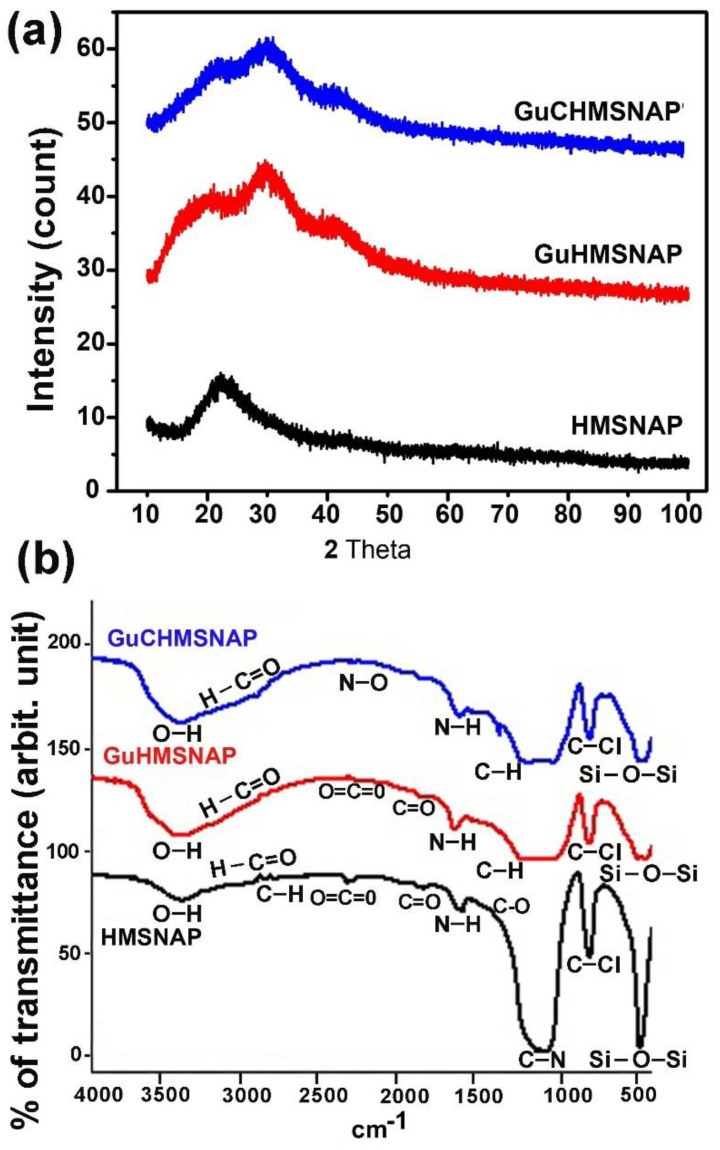
XRD (**a**) and FTIR (**b**) spectrum of HMSNAP and drug-loaded HMSNAP.

XRD analysis exposed that HMSNAP and drug-loaded HMSNAP are amorphous in nature because of the silica material, deposition of drugs, and APTES lesion found in HMSNAP at 6–37 of two degrees theta (Figure 2a). FTIR analysis with a wavelength range from 500 to 1800 cm^−1^ proved to be the proximity functional group of Si-O-Si, C-Cl, C-H, N-H, and C=O (Figure 2b). The O=C=O, N-O, H-C=O, and –OH peaks from 2200 to 3780 cm^−1^ illustrate that guanidine and curcumin were loaded on HMSNAP. Finally, massive discrepancies were recorded in Gu-HMSNAP and GuC-HMSNAP when compared with HMSNAP. The peak range of 2943 cm^−1^ showed the proximity of the amine group and the –C-H group peak range of 2830 cm^−1^, which specifies the functional group APTES. As a whole, our characterisation studies clearly demonstrate that guanidine and curcumin are stuffed with the hollow and mesopore of HMSNAP, which are encouraged to modify carrier size, nanopores, surface charges, and modified functional groups. This proves that significant amounts of drugs can be loaded onto nanocarriers for breast cancer therapy.

### 3.4. XPS analysis of drug-loaded HMSNAP

**Figure 3:**
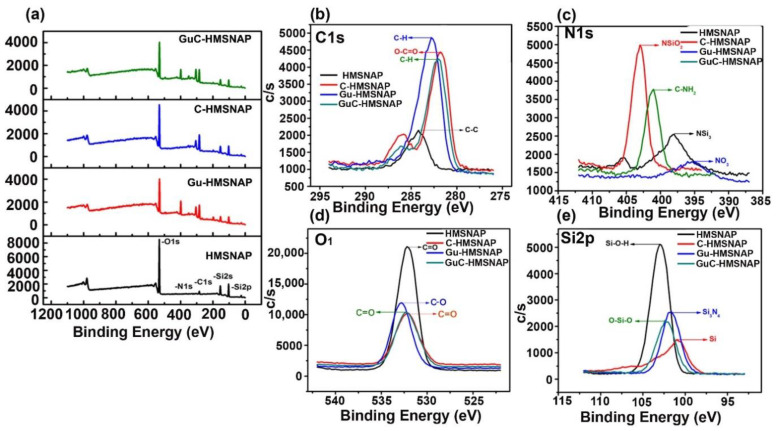
The XPS spectra of survey (**a**) and high resolution (**b**–**e**) of HMSNAP and drug-loaded HMSNAP. The region of C1s (**b**), N1s (**c**), O1s (**d**), and Si2p (**e**).

XPS was used to examine the surface elemental analysis of HMSNAP and drug-loaded HMSNAP. The elements Si, O, C, and N were detected in HMSNAP, Gu-HMSNAP, C-HMSNAP, and GuC-HMSNAP at the range of 0 to 1100 eV in the XPS spectrum, as shown in Figure 3. The major elements Si and O developed from the hydrolysis of TEOS, whereas the APTES coating confirms the presence of C and N. In addition, the maximum percentage of C was evolved in the drug-loaded sample of C-HMSNAP, Gu-HMSNAP, and GuC-HMSNAP, which effectively established the surface modification of HMSNAP. In addition, the Si and O peak intensity decreased after surface modification with guanidine and curcumin loading. Furthermore, the XPS results demonstrate that we successfully modified HMSNAP alone and in combination with guanidine and curcumin.

In this experiment, the survey spectrum of peak intensity on Si2p/2s in HMSNAP, Gu-HMSNAP, C-HMSNAP, and GuC-HMSNAP are shown in Figure 3a; binding energy 102.1 to 102.9 ev confirmed the presence of Si2p/2s. The high-resolution XPS spectra of C1s, N1s, O1s, and Si2p/2s are shown in Figure 3b–e. The intensity of Si2p/2s in HMSNAP was more than 40.00% when compared with drug-loaded HMSNAP. The intensity of the O1 spectrum in the ranges of 532.1 to 532.8 eV is shown in Figure 3d; the peak intensity of drug-loaded HMSNAP slightly decreased when compared with HMSNAP. The ranges 398 to 402.2 eV and 282 to 284.2 eV confirm the spectrum of N1s and C1s, respectively. The sharp peak intensity of N1s and blunt C1s intensity peak gradually increased because of the presence of curcumin and guanidine in HMSNAP. 

The percentages of the elements of HMSNAP and drug-loaded HMSNAP were calculated using XPS spectra, as shown in Table 1. The percentage of the elements Si, N, O, and C in HMSNAP were 25.6, 0.4, 65.8, and 8.2%, respectively. Those of N, O, and C were slightly increased in drug-loaded HMSNAP when compared with HMSNAP. The percentage of elements of Si, N, O, and C in Gu-HMSNAP were 14.60, 11.10, 33.10, and 41.20%, respectively. The immobilisation of APTES on the surface of HMSNAP through hydrolysis reaction confirmed the increasing percentage of N. Comparatively, the percentage of elements in C-HMSNAP was Si (10.4%), N (8.9%) O (33%), C (47.40%), while GuC-HMSNAP contained Si (12.50%), N (14.40%), 0 (37.70%), and C (35.50%). The guanidine and curcumin binding confirms that the expression of C in Gu-HMSNAP, C-HMSNAP and GuC-HMSNAP dramatically increased when compared to HMSNAP.

### 3.5. N_2_ Adsorption-Desorption Isotherms of HMSNAP and Drug-Loaded HMSNAP

**Figure 4:**
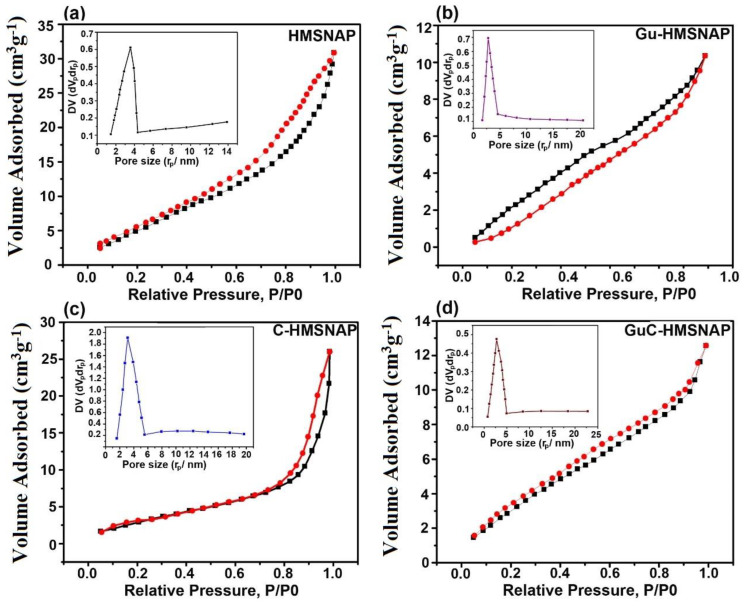
Nitrogen adsorption-desorption isotherms and pore size distribution (inset figure) of HMSNAP (**a**), Gu-HMSNAP (**b**), C-HMSNAP (**c**), and GuC-HMSNAP (**d**).

The surface area, pore volume, and N2 adsorption-desorption isotherms of HMSNAP and drug-loaded HMSNAP were analysed, and are shown in Figure 4, whereas the mesoporous structure was explained based on a hysteresis H1-type loop [42]. The relative pressure (P/P^0^) ranged from 0.2–1.0, confirming the monolayer absorption of HMSNAP and drug-loaded HMSNAP [43]. The respective relative pressure P/P^0^ of HMSNAP, Gu-HMSNAP, C-HMSNAP, and GuC-HMSNAP were 0.8, 0.3, 0.8 and 0.2. (Figure 4a–d). The shape of the hysteresis loop was changed for drug−loaded HMSNAP and HMSNAP, which clearly indicates that the pore volume, size, and absorption was altered due to the curcumin and guanidine loaded in the nanocarrier. Table 2 shows that the Brunauer–Emmett–Teller (BET) surface area and pore volume of HMSNAP was 969.78 m^2^ g^−1^ and 2.71 cm^3^ g^−1^, respectively. The BET surface area was 750.06 m^2^ g^−1^ and 830.66 m^2^ g^−1^, and the pore volume was 1.89 cm^3^ g^−1^ and 2.38 m^2^ g^−1^, respectively, for separately guanidine-loaded and curcumin-loaded HMSNAP. Therefore, the drug-loaded HMSNAP surface area and pore volume were reduced when compared with HMSNAP.

Furthermore, the surface area and pore volume of guanidine–curcumin complex-loaded HMSNAP was 657.39 m^2^ g^−1^ and 1.14 m^2^ g^−1^, respectively. The surface area and pore volume of both drug-loaded nanocarriers were reduced when compared with the single drug loaded in HMSNAP. The functionalisation of APTES in guanidine and carboxylation of curcumin provides effective evidence for the maximum amount of drug loading in HMSNAP. Similarly, the pore diameters of drug-loaded HMSNAP were 2.74, 3.10 and 2.52 for Gu-HMSNAP, C-HMSNAP and GuC-HMSNAP, respectively, whereas the pore diameter of HMSNAP was 3.56. Therefore, the pore diameter was reduced in drug-loaded HMSNAP when compared with HMSNAP, as shown in Figure 4. Therefore, more than 40.00% of pore diameters and 50.00% of pore volumes were masked with guanidine and curcumin in HMSNAP. The HMSNAP and drug-loaded HMSNAP pore diameter provides sufficient evidence for the binding of the drugs with a similar nanocarrier, while the structure of mesoporous silica remained intact.

### 3.6. NMR Analysis of Drug-Loaded HMSNAP

**Figure 5:**
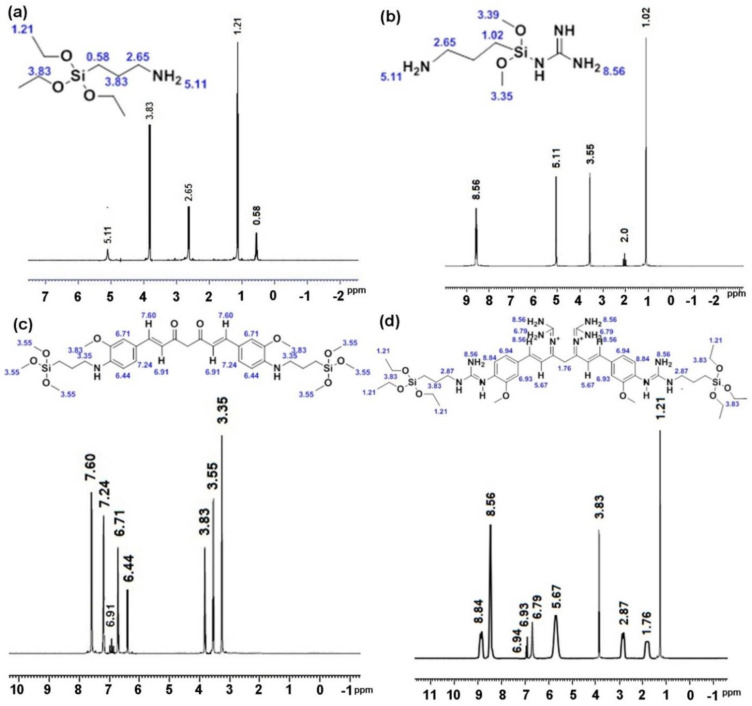
^1^H NMR spectrum of HMSNAP (**a**), Gu-HMSNAP (**b**), C-HMSNAP (**c**), and GuC-HMSNAP (**d**).

NMR is a promising technique used to determine the structural and functional groups of high biomolecules. In NMR spectral analysis, dimethyl sulfoxide (DMSO) is the most commonly used solvent due to its polarity. HMSNAP and drug-loaded HMSNAP were investigated through the ^1^H NMR spectrum, as shown in Figure 5a. In HMSNAP, ppms of 0.58, 2.65 (–O-Si) represent the functional group of silica, and the immobilisation of APTES with a silica surface is confirmed at the ppm of 5.11 (N-H). Hence, the guanidine loading on the nanocarrier and the chemical shift is confirmed in the range of 2.0 and 8.56. In addition, the chemical shift range of 5.11 confirms that APTES is masked with guanidine-loaded nanoparticles (Figure 5b). After that, the aromatic group of curcumin (O-C) binds with the surface of silica (-O-Si) through methine group formation and carboxylation at the range of 3.35, 3.55, and 3.83, respectively. Furthermore, the ppm ranges of 6.44, 6.71, 6.91, 7.24, and 7.60 confirm the presence of curcumin inside the nanocarrier (Figure 5c). Finally, the guanidine–curcumin complex formation is confirmed via the amine group alteration in curcumin at the range of 8.56 and 8.84. The APTES in the surface of silica directly binds with the amine group of guanidine in the range of 2.87 and 3.83, which confirms the guanidine–curcumin nano-complex (Figure 5d). 

The nanocarrier HMSNAP strongly binds alone and with the complex of guanidine, curcumin, and guanidine-curcumin. The guanidine-curcumin nano-complex indicates that the intensity of guanidine is marginally higher than the guanidine nano-complex. Overall, the NMR results suggest that the single and complex drugs are loaded in nanoparticles through modification in surface and functional groups. Thus, the maximum amounts of drugs can be loaded into the HMSNAP for the therapeutic purpose of breast cancer.

### 3.7. Guanidine and Curcumin Loading and Release in HMSNP and HMSNAP

**Figure 6:**
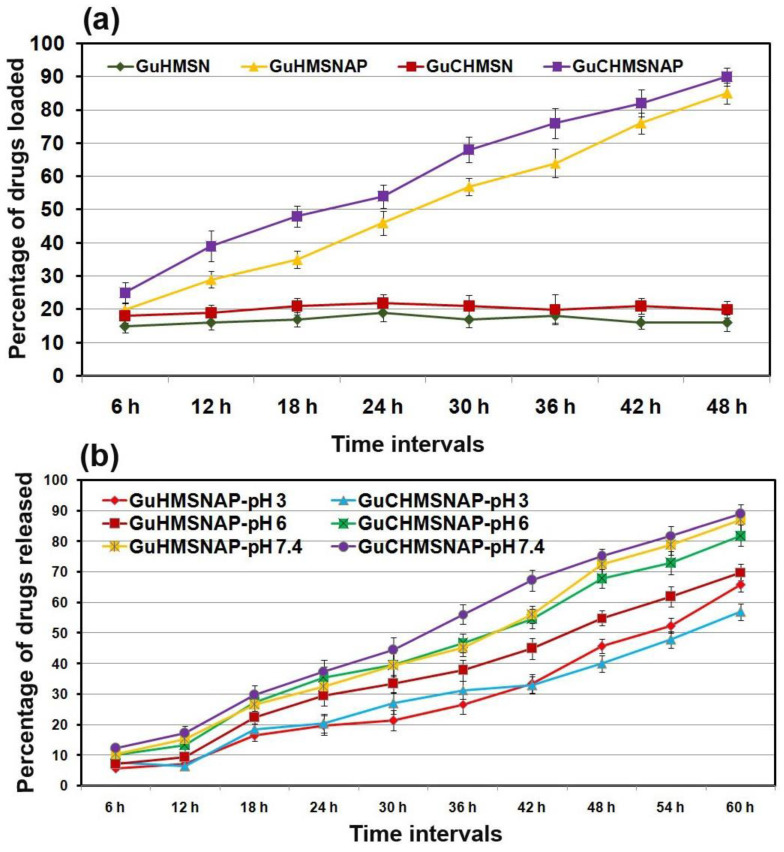
The guanidine and guanidine-curcumin complex loading into HMSNP and HMSNAP at different time intervals (**a**) and guanidine and guanidine-curcumin complex released from HMSNAP in a pH- and time-dependent manner (**b**).

The guanidine and the combination of guanidine and curcumin were dissolved separately in ethanol (100%). Later, these drugs were independently diffused with HMSNP and APTES-decorated HMSNAP and their drug encapsulation ability was appraised, as shown in Figure 6a. The aggregate of guanidine and guanidine-curcumin complexes as cargo with amine-functionalised HMSNAP was determined by loosening drugs available in a flask, and was estimated at every 6 h intervals and up to 48 h of incubation. The guanidine-curcumin complex as cargo with HMSNAP was reported as 90%, though 20% of this was loaded in HMSNP. Thus, APTES is a well-organised encapsulation with silica nanoparticles and can provide a rich amine group on the surface of the mesosphere shell. APTES is able to provide an intermolecular hydrogen bond between the receptor of drugs and the surface of the hollow mesosphere. The guanidine-curcumin complex has five hydrogen donors and five hydrogen acceptors. Therefore, APTES can link efficiently with drugs and the mesosphere [40]. The cargo of guanidine-curcumin complex with nanocarriers develops by either hydrogen bonding or electrostatic interactions, which both have a notably increased loading ability [44].

The guanidine-curcumin complex as cargo with HMSNAP has an estimated drug release at acidic pH 3.0, mild acidic 6.0, and normal pH 7.4 with phosphate buffer saline, as shown in Figure 6b. The drugs are slowly and steadily released from the mesoporous nanoparticles depending on the prolonged incubation time and pH. The guanidine-curcumin complex is released at 90% at pH 7.4, 85% at pH 6.0, and 60.40% at pH 3.0 at 60 h. Likewise, 86, 73.7, and 65.30% of guanidine is released at pH 7.4, 6.0, and 3.0 at 60 h, respectively. The changes in pH slightly alter the release of guanidine-curcumin complex and guanidine from HMNSAP. In general, PBS at pH 7.4 is commonly used to mimic the colon. However, the guanidine-curcumin complex is released successfully at a neutral pH compared with acidic pH ranges. These results depict a huge volume of drug molecules is retained in the reservoir of the mesoporous shell and released steadily in a pH- and time-dependent manner. The amount of drugs released from HMNSAP depends on the degradability of the APTES coating. A large amount of drugs are released at pH 7.4 because an ethoxy group of APTES is hydrolysed at a higher pH range [44].

### 3.8. Drug Release Kinetics

The in vitro drug release kinetics data on both guanidine and guanidine-curcumin complex-loaded formulations are presented in Table 3. The regression coefficient (*r*^2^) value and release rate constant were predicted for each kinetic model. In general, the nearer the regression coefficient value (*r*^2^) to 1, the greater the fit or relationship between the two factors. The release design of zero-order kinetics regression coefficient values (*r*^2^) is much nearer to 1 for both experiments, i.e., for guanidine 0.9442 (pH 3), 0.9797 (pH 6.0), 0.9834 (pH 7.4), and curcumin 0.9638 (pH 3.0), 0.9852 (pH 6.0), and 0.9865 (pH 7.4) (source file attached in Supplementary Table S1). Except for the Korsmeyer-Peppas model, all of the models evaluated had an *r*^2^ value greater than 0.96. The attained findings on the guanidine and curcumin release kinetics indicate that the transport mechanism was stimulated by Fickian diffusion. The controlled release of guanidine and curcumin from HMSN was adapted with Fickian diffusion. The zero-order kinetics were recommended as the most efficient model for the guanidine and curcumin release mechanism based on these kinetic experiments. This illustrates the nanoparticle formulation’s homogeneous dissolution and regulated release.

### 3.9. Cytotoxicity of HMSNAP, Gu-HMSNAP, and GuC-HMSNAP 

**Figure 7:**
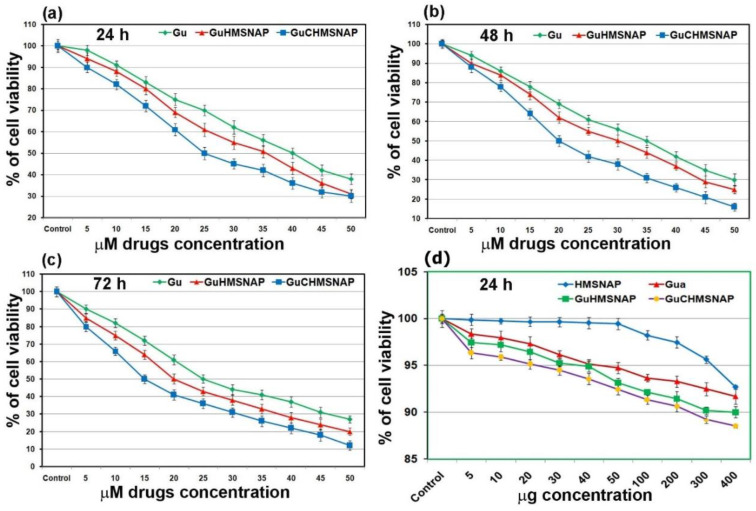
The cell viability assessment of different concentrations of guanidine alone, guanidine-loaded HMSNAP, and guanidine-curcumin complex-loaded HMSNAP-treated MCF-7 cells. Dose and time-dependent manner of cell viability testing with free guanidine, Gu-HMSNAP, and GuC-HMSNAP-treated MCF-7 cells at 24 h (**a**), 48 h (**b**), and 72 h (**c**) and HMSNAP, guanidine alone, guanidine-loaded HMSNAP, and guanidine-curcumin complex-loaded HMSNAP at 24 h (**d**). The cell viability of HEK cells is expressed as a percentage related to untreated cells, and is set as 100%.

Identification of IC_50_ values of free guanidine, Gu-HMSNAP, and GuC-HMSNAP were treated with HEK 293 (Supplementary Figure S1) and MCF-7 cells through cell death as shown in Figure 7. APTES-decorated HMSNAP produced lesser toxicity in HEK 293 and MCF-7 cells. Hence, 2 to 6 µg of nanocarrier were adopted for loading of guanidine and guanidine-curcumin in these experiments [45]. In the cytotoxicity assay, 30 µM of curcumin induced 50% of cell death in MCF-7 [19,46] and the IC_50_ values of free guanidine, Gu-HMSNAP, and GuC-HMSNAP were 40, 35, and 25 µM, respectively, at 24 h (Figure 7a). Therefore, cell death induced by guanidine alone and by guanidine–curcumin complex were directly correlated in a dose- and time-dependent manner. After 48-h incubation with MCF-7 cells, free guanidine 35 µM, Gu-HMSNAP 30 µM, and GuC-HMSNAP 20 µM induced 50% cell death (Figure 7b). At 72 h incubation, 25 µM guanidine, 20 µM Gu-HMSNAP, and 15 µM GuC-HMNSAP induced 50% cell death (Figure 7c). Hence, the IC_50_ value of guanidine–curcumin complex cargo with nanocarrier was decreased the amount of guanidine by 50% compared with the free guanidine concentration. Thus, the combination of guanidine–curcumin complex cargo with a silica nanosphere efficiently induced apoptosis at a minimal guanidine concentration.

### 3.10. Evaluation of Apoptosis in Guanidine-Curcumin Complex-Loaded HMSNAP on MCF-7 Cells

**Figure 8:**
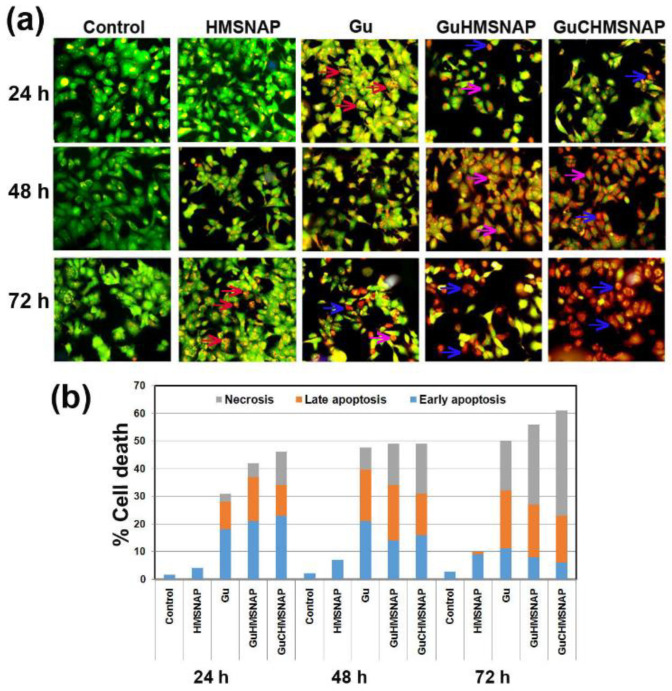
Photomicrographs of acridine orange displaying HMSNAP and drug-loaded HMSNAP-induced cell death in MCF-7 cells at different time intervals. The arrow marked with a blue colour represents early apoptosis, the orange colour represents late apoptosis, and the gray colour represents necrosis of MCF-7 cells. (**a**) The graph represents the percentage of early and late apoptosis and necrosis in HMSNAP and drug-loaded HMSNAP-induced cell death in MCF-7 cells at different time intervals (**b**).

The main focus of developing anticancer drugs is inducing effective cancerous cell death, and GuC-HMSNAP is an efficient curative medicine and activator of apoptosis in cancer cells. In this regard, GuC-HMNSAP-treated cells with modified morphological changes were estimated through acridine orange/ethidium bromide dual staining (Figure 8a). The control and HMNSAP-treated MCF-7 cells display green fluorescence and homogenous round nuclei, which indicates viable cells. IC_50_ value concentrations of free guanidine, Gu-HMNSAP, and GuC-HMNSAP-treated cells at 24 h incubation presented an orange colour, cell shrinkage, and nuclear condensation, which indicates the early stage of apoptosis. In addition, the Gu-HMNSAP and GuC-HMNSAP-treated cells at both 48 and 72 h were found in red colour fluorescent cells containing apoptotic bodies, which indicates necrotic cells and complete loss of membrane integrity. The early apoptosis, late apoptosis, and necrosis percentages of drug-treated MCF-7 cells are shown in Figure 8b. HMSNAP-treated cells showed that 3 to 10% of cell death occurred within 24 to 72 h of incubation. The free guanidine, Gu-HMNSAP, and GuC-HMNSAP-treated cells led to cell death from 31 to 49, 42 to 55, and 46 to 61, respectively, from 24 to 72 h. Late apoptosis was found more in drugs incubated at a 48 h incubation interval than at a 24 h interval. Necrosis gradually increased in a time-dependent manner, and significant necrosis was found with Gu-HMSNAP (16.50%) and GuC-HMSNAP (19.20%) at 48 h and with free guanidine (18.42%), Gu-HMSNAP (27.84%), and GuC-HMSNAP (36.34%) at 72 h. The guanidine results demonstrate efficient induction of cell death in various cancerous cell lines without similar action against normal cells [47]. The side group of guanidine contributes a variety of applications, including identifying receptors by non-covalent interactions such as hydrogen bonding and electrostatic binding. The guanidine linkage appears to be bound with nuclease, then turned into a positively charged enzyme, giving rise to increased cell membrane permeability via electrostatic attraction and induced cancer cell death.

### 3.11. Gu-HMSNAP and GuC-HMSNAP Inactivation of Phosphorylation of Tumourigenic Proteins 

**Figure 9:**
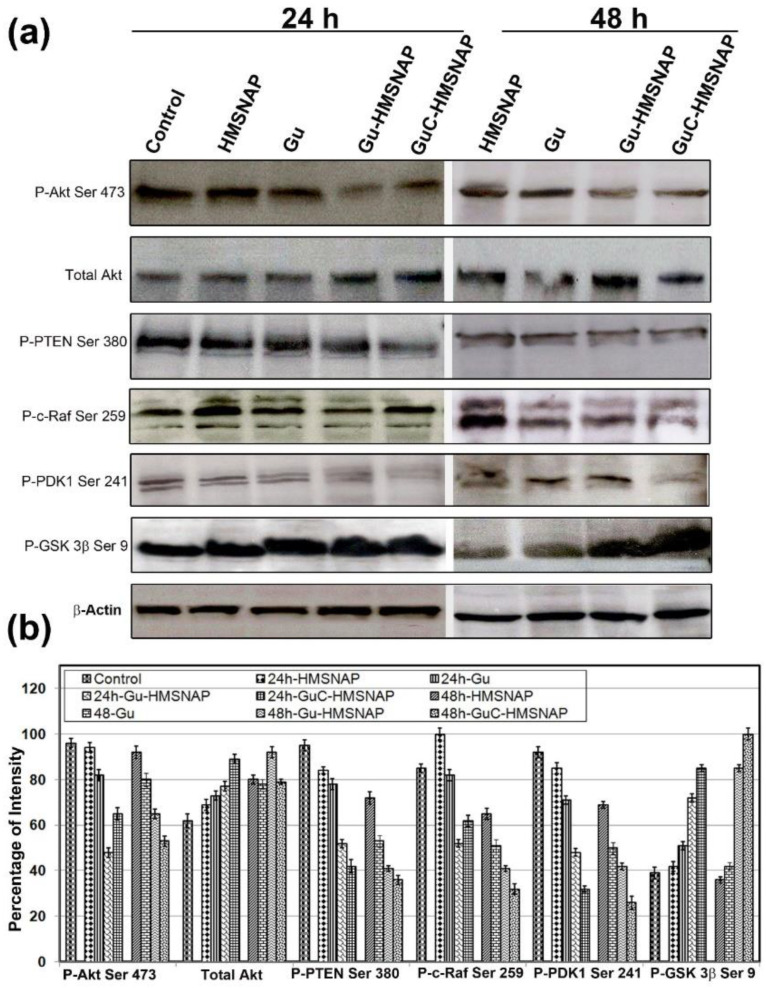
Western blot analysis was used to determine the phosphorylation of oncoproteins and tumour-suppressing proteins in MCF-7 cells treated with HMSNAP and drug-loaded HMSNAP at 24 h and 48 h incubation. (**a**) The graph represents the densitometric analysis of each protein as compared with the respective control and calculates the percentage manipulation (**b**).

The altered apoptosis with the guanidine carbonate-curcumin complex was analysed through tumour-inducing and tumour-suppressing proteins in MCF-7 cells (Figure 9). The phosphorylation of Akt S473 was downregulated approximately two-fold in both 24 h and 48 h intervals of Gu-HMSNAP and GuC-HMSNAP-treated MCF-7 cells when compared with the control. The total Akt was gradually altered by 0.5% in drug-loaded nanocarriers. The phosphorylation of PTEN at S380 in free guanidine carbonate, Gu-HMSNAP, and GuC-HMSNAP-treated cells were reduced approximately two-fold after 24 h and three-fold after 48 h compared with the control cells (Figure 9). The downregulation of PTEN in MCF-7 cells induced cell death through inactivation of phosphorylation of Akt Ser473, then through activation of ATP and caspases [48]. Phosphorylation of c-Raf Ser249 was downregulated approximately two-fold after 24 h and three-fold after 48 h in GuC-HMSNAP-treated cells. C-Raf was found in the plasma membrane and mitochondrial membrane followed by downregulation of Akt through phosphorylation at Ser259 of c-Raf, and cell death was induced [49]. Phosphorylation of GSK-3β Ser9 in Gu-HMSNAP-treated and GuC-HMSNAP-treated MCF-7 cells demonstrated an approximately two-fold increase in both time intervals when compared with control. GSK-3β plays a major role in the regulation of the cell cycle through cyclin D1 phosphorylation. The phosphorylation of Ser9 inactivates the GSK-3β. Therefore, our results suggest that guanidine carbonate–curcumin complexes cause downregulation of phosphorylation in Ser471 of Akt, Ser259 of c-Raf, and Ser241 of PDK1 and upregulation of phosphorylation in GSK-3β Ser9, which then partially induces intrinsic cell death in MCF-7 [50].

### 3.12. GuC-HMSNAP Induces Apoptotic Proteins

**Figure 10:**
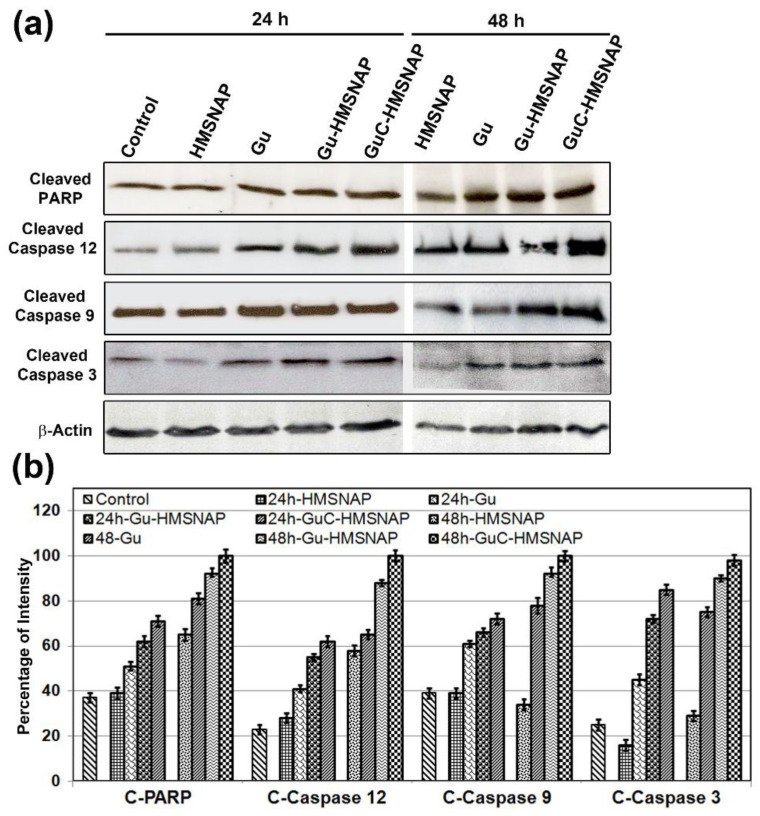
Western blot analysis of apoptotic proteins in MCF-7 cells treated with HMSNAP and drug-loaded HMSNAP at 24 h and 48 h intervals (**a**). The graph represents the densitometric analysis of each protein compared with the respective control and calculates the percentage manipulation (**b**).

The nuclear protein PARP is involved in the repair of damaged DNA, and is chopped by caspases to form cleaved PARP, which induces apoptosis due to inability to repair DNA damage. Western blot analysis of c-PARP, cleaved caspase 12, cleaved caspase 9, and cleaved caspase 3 showed approximately two-fold upregulation in 24 h intervals and three-fold upregulation in 48 h intervals in Gu-HMSNAP-treated and GuC-HMNSAP-treated MCF-7 cells in comparison with the respective controls (Figure 10). Cleaved caspases and cleaved PARP are considered a hallmark of apoptosis [51,52]. Gu-HMSNAP and GuC-HMNSAP induced cell death by promoting c-PARP. HMSNAP-treated cells showed no significant changes compared with control cells, and were observed in all apoptotic proteins. Therefore, minimal concentrations of HMSNAP are non-toxic and do not alter any of the apoptotic proteins.

## 4. Conclusions

The current study focused on evaluating the co-delivery efficacy of guanidine carbonate and guanidine–curcumin complex cargo with HMSNAP, which induces MCF-7 cell death. Characterisation studies clearly demonstrate the modification of mesosphere size and nanopores, surface charges, surface area, pore volume, drug complex analysis, and changes in functional groups. In addition, a significant amount of C and N were found in the drug-loaded HMSNAP. On the other hand, O and Si were drastically reduced in the drug-loaded nanocarriers when compared with HMSNAP. Furthermore, pore volume and pore diameter were reduced, confirming that the drugs loaded in the nanocarrier. Altogether, significant amounts of drugs can be carried as cargo by the nanocarrier for breast cancer therapy. The docking studies clearly demonstrate that the guanidine–curcumin complex is associated with tumour-suppressing proteins through hydrogen bonding and electrostatic interactions with lower binding affinity, ultimately leading to cancer cell death. Western blot analysis illustrated that GuC-HMSNAP is associated with oncoproteins, then induces the intrinsic cell death pathway. As a whole, our results clearly illustrate that GuC-HMNSAP is an efficient nanocarrier with a potentially huge volume of cargo molecules, altered surface charges, and functional groups, and which can efficiently induce cancer cell death.

## Figures and Tables

**Table 1:** The percentage of Si, N, O, Ga, and C of HMSNAP and drug-loaded HMSNAP based on XPS results.

Nanoparticles	Silica	Nitrogen	Oxygen	Carbon
HMSNAP	25.6	0.4	65.8	8.2
C-HMSNAP	10.4	8.9	33.0	47.7
Gu-HMSNAP	14.6	11.1	33.1	41.2
GuC-HMSNAP	12.5	14.4	37.7	35.5

**Table 2:** Textural properties of HMSNAP and drug-loaded HMSNAP.

Sample	Surface Area (m^2^ g^−1^) S_BET_ (m^2^ g^−1^)	Pore Diameter (nm) D_pDES_ (nm)	Pore Volume (cm^3^ g^−1^) V_p_ (cm^3^ g^−1^)
HMSNAP	969.78	3.56	2.71
C-HMSNAP	830.66	3.10	2.38
Gu-HMSNAP	750.06	2.74	1.83
GuC-HMSNAP	657.39	2.52	1.14

**Table 3:** Drug release kinetics of HMSNAP and drug-loaded HMSNAP.

Parameters		Guanidine-Loaded HMSNAP			Guanidine Curcumin-Loaded HMSNAP		
		pH 3.0	pH 6.0	pH 7.4	pH 3.0	pH 6.0	pH 7.4
Zero-order F = K_0_ × t	K_0_	0.016	0.020	0.024	0.016	0.024	0.027
	*r* ^2^	0.94	0.97	0.98	0.96	0.98	0.98
	AIC	54.18	45.66	48.42	46.39	45.86	46.78
First-order F = 100 × [1 − Exp (−K_1_ × t)]	K_1_	0.00	0.00	0.00	0.00	0.00	0.00
	*r* ^2^	0.88	0.95	0.90	0.94	0.93	0.93
	AIC	61.81	54.54	65.64	50.48	60.60	61.74
Higuchi model F = KH × t1/2	KH	0.75	0.96	1.20	0.78	1.18	1.31
	*r* ^2^	0.70	0.82	0.80	0.82	0.83	0.86
	AIC	70.79	67.29	72.93	62.06	69.71	70.04
Korsmeyer–Peppas model F = kKP × t^n^	kKP	0.001	0.029	0.025	0.03	0.079	0.068
	*r* ^2^	0.97	0.98	0.98	0.96	0.98	0.99
	n	1.30	0.95	0.99	0.91	0.84	0.88
	AIC	49.55	46.97	50.41	47.31	45.29	37.65
Hixon–Crowell model F = kF = 100 × [1 − (1 − kHC × t)3]KP × tn	kHC	0.00	0.00	0.00	0.00	0.00	0.00
	*r* ^2^	0.90	0.96	0.94	0.95	0.96	0.97
	AIC	59.76	50.46	61.01	48.45	54.97	53.54

AIC = Akaike information criterion, F = fraction of drug release in time t, K_0_ = apparent rate constant of zero order release constant, K_1_ = first order release constant, KH = Higuchi constant, kKP = Korsmeyer–Peppas rate constant, kHC = Hixon–Crowell constant, n = diffusional exponent. And r^2^ = Squared correlation coefficient.

## Data Availability

The original contributions presented in the study are included in the article. Further inquiries can be directed to the corresponding author(s).

## Supplementary Materials

The following supporting information can be downloaded at: https://www.mdpi.com/article/10.3390/cancers14143490/s1. Figure S1. The cell viability assessment of different concentrations of guanidine alone, guanidine-loaded HMSNAP, and guanidine-curcumin complex-loaded HMSNAP-treated HEK 293 cells. Dose- and time-dependent manner of cell viability testing with free guanidine, Gu-HMSNAP, and GuC-HMSNAP-treated MCF-7 cells at 24 h (a), 48 h (b), 72 h (c), and HMSNAP, guanidine alone, guanidine-loaded HMSNAP, and guanidine–curcumin complex-loaded HMSNAP at 24 h (d). Results of MTT are expressed as a percentage related to untreated cells and are set as 100%. Table S1: Drug release kinetics studies source file.
