# Supplementary material for: Guanidine–Curcumin Complex-Loaded Amine-Functionalised Hollow Mesoporous Silica Nanoparticles for Breast Cancer Therapy
Source: Cancers (Basel). 2022 Jul 18;14(14):3490. doi: 10.3390/cancers14143490 (PMC9323383; doi:10.3390/cancers14143490)
Supplement: Supplementary file 1 [file cancers-14-03490-s001.zip › cancers-1796436 - Supplementary Figure S1.pdf]

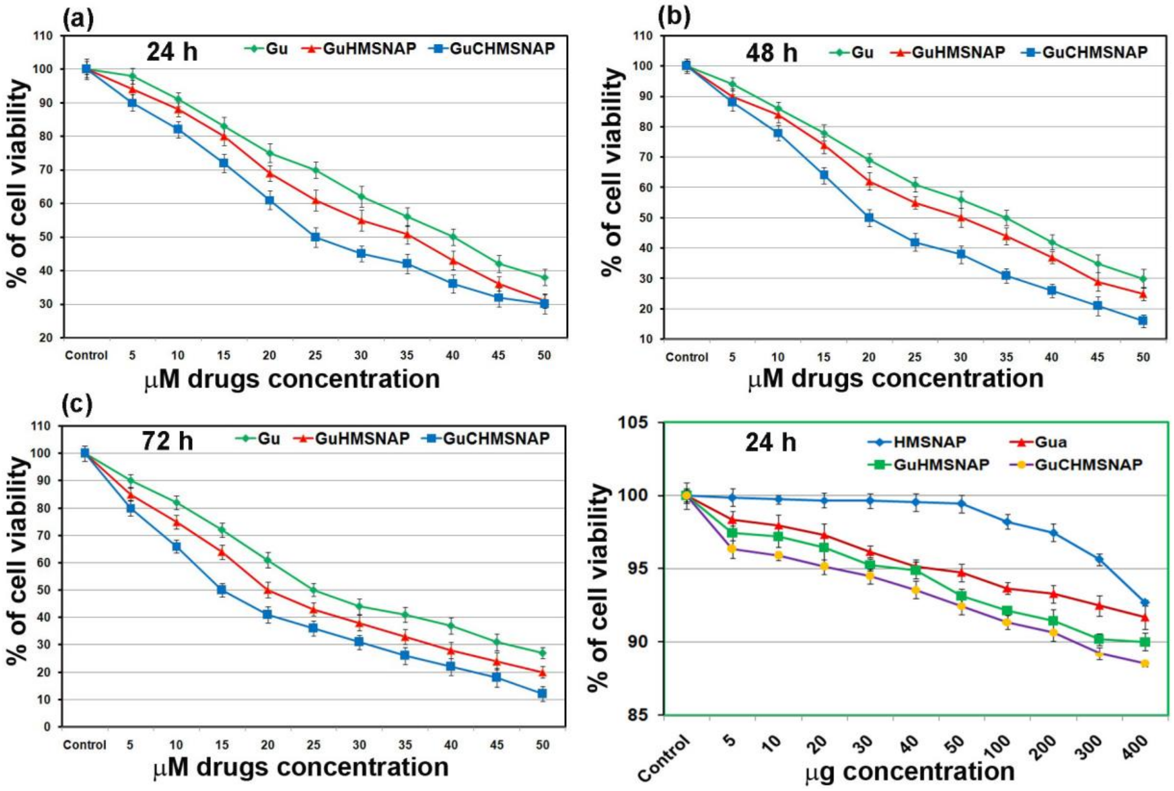

Figure S1. The cell viability assessment of different concentrations of guanidine alone, guanidine-loaded HMSNAP and guanidine curcumin complex-loaded HMSNAP-treated HEK 293 cells. Dose and time-dependent manner of cell viability testing with free guanidine, Gu-HMSNAP, and GuC-HMSNAP-treated MCF-7 cells at 24 h (**a**), 48 h (**b**), 72 h (**c**) and HMSNAP, guanidine alone, guanidine-loaded HMSNAP and guanidine curcumin complex-loaded HMSNAP at 24 h (**d**). Results of MTT are expressed as a percentage related to untreated cells and are set as 100%.
